# Feasibility of 3D Body Tracking from Monocular 2D Video Feeds in Musculoskeletal Telerehabilitation

**DOI:** 10.3390/s24010206

**Published:** 2023-12-29

**Authors:** Carolina Clemente, Gonçalo Chambel, Diogo C. F. Silva, António Mesquita Montes, Joana F. Pinto, Hugo Plácido da Silva

**Affiliations:** 1 Instituto Superior Técnico (IST), Department of Bioengineering (DBE), Av. Rovisco Pais n. 1, 1049-001 Lisboa, Portugal; 2 CLYNXIO, LDA, Rua Augusto Macedo, n. 6, 5 Dto., 1600-794 Lisboa, Portugal; 3 Department of Physiotherapy, Santa Maria Health School, Trav. Antero de Quental 173/175, 4049-024 Porto, Portugal; diogo.silva@santamariasaude.pt (D.C.F.S.); antonio.montes@santamariasaude.pt (A.M.M.); 4 Department of Functional Sciences, School of Health, Polytechnic Institute of Porto, Rua Dr. António Bernardino de Almeida 400, 4200-072 Porto, Portugal; 5 Center for Rehabilitation Research, School of Health, Polytechnic Institute of Porto, Rua Dr. António Bernardino de Almeida 400, 4200-072 Porto, Portugal; 6 Department of Physiotherapy, School of Health, Polytechnic Institute of Porto, Rua Dr. António Bernardino de Almeida 400, 4200-072 Porto, Portugal; 7 Instituto de Telecomunicações (IT), Av. Rovisco Pais n. 1, Torre Norte—Piso 10, 1049-001 Lisboa, Portugal; 8 Lisbon Unit for Learning and Intelligent Systems (LUMLIS), European Laboratory for Learning and Intelligent Systems (ELLIS), Av. Rovisco Pais n. 1, 1049-001 Lisboa, Portugal

**Keywords:** telerehabilitation, musculoskeletal, 3D Human Pose Estimation, MediaPipe Pose, ROM, 2D camera, monocular, videos, deep learning

## Abstract

Musculoskeletal conditions affect millions of people globally; however, conventional treatments pose challenges concerning price, accessibility, and convenience. Many telerehabilitation solutions offer an engaging alternative but rely on complex hardware for body tracking. This work explores the feasibility of a model for 3D Human Pose Estimation (HPE) from monocular 2D videos (MediaPipe Pose) in a physiotherapy context, by comparing its performance to ground truth measurements. MediaPipe Pose was investigated in eight exercises typically performed in musculoskeletal physiotherapy sessions, where the Range of Motion (ROM) of the human joints was the evaluated parameter. This model showed the best performance for shoulder abduction, shoulder press, elbow flexion, and squat exercises. Results have shown a MAPE ranging between 14.9% and 25.0%, Pearson’s coefficient ranging between 0.963 and 0.996, and cosine similarity ranging between 0.987 and 0.999. Some exercises (e.g., seated knee extension and shoulder flexion) posed challenges due to unusual poses, occlusions, and depth ambiguities, possibly related to a lack of training data. This study demonstrates the potential of HPE from monocular 2D videos, as a markerless, affordable, and accessible solution for musculoskeletal telerehabilitation approaches. Future work should focus on exploring variations of the 3D HPE models trained on physiotherapy-related datasets, such as the Fit3D dataset, and post-preprocessing techniques to enhance the model’s performance.

## 1. Introduction

Musculoskeletal conditions are the leading cause of physiotherapy demand globally; it is estimated that approximately 1.71 billion people have a musculoskeletal condition worldwide, and its prevalence is expected to increase [1]. Conventional musculoskeletal rehabilitation typically involves multiple in-clinic sessions, high travelling and waiting times, little schedule flexibility and complementary home exercises, becoming inconvenient and expensive for both patients and clinics [2]. Consequently, providing quality care to a patient at a distance (i.e., telerehabilitation) through digital and gamified solutions has become a topic of growing interest [2].

The development of telerehabilitation has increased in recent years, due to factors including technological innovation, the impact of the COVID-19 pandemic, and the rising concern about patient-centered treatment [3,4]. Telerehabilitation has been demonstrated to offer enhanced accessibility, affordability, personalization, and real-time monitoring; furthermore, it empowers patients to take charge of their well-being while ensuring convenience, adherence, and motivation [3,5]. Moreover, by reducing wait times, facilitating long-term management, and fostering patient education, telerehabilitation is emerging as an indispensable component of modern healthcare, reshaping the future of physiotherapy services [4]. Musculoskeletal telerehabilitation should resemble traditional sessions and allow for equivalent outcomes compared to conventional methods, in order to ensure its widespread acceptance and integration into healthcare systems [3]. Human body tracking is, therefore, a key element, since it allows physiotherapists to be aware of the movement and performance of their patients during the physiotherapy session [6], while following the relevant clinical and motion information, such as the Range of Motion (ROM) of the human joints. The joint ROM is defined as “the amount of movement that occurs at a joint to produce movement of a bone in space”, i.e., the angle range of a joint during an exercise [7]. It is a valuable musculoskeletal metric since it provides information to identify limitations and imbalances in joint movement, gives useful knowledge for developing an appropriate treatment, and helps to ensure that exercises are being performed correctly and safely, maximizing the benefits of the exercise and minimizing the risk of injury [7].

Human body tracking systems for musculoskeletal rehabilitation purposes include optical marker-based systems [8], wearables [9,10], markerless 3D or RGB-D (Red Green Blue-Depth) camera-based systems [5,11], and markerless 2D or RGB (Red Green Blue) camera-based systems [12].

Optical marker-based and wearable sensor systems provide highly accurate body tracking but can be expensive and cumbersome [13,14]. On the other hand, 3D and 2D camera-based systems are more convenient for patients as no body-worn markers/sensors are required (markerless); however, 3D cameras may be costly when compared to 2D cameras, often have software restrictions (e.g., Orbbec Astra 3D cameras are incompatible with MacOS X), and depend on calibration and luminosity conditions [15]. Two-dimensional camera-based systems are a promising, accessible, and affordable body tracking approach [16]. These only require images or videos from a regular 2D camera (available in most standard computers and smartphones) to reconstruct the human body, typically through deep learning models [12]. Considering the advantages of 2D camera-based systems, this work aims to evaluate if these approaches are feasible for musculoskeletal telerehabilitation purposes. To the best of our knowledge, this is the first work investigating a 2D camera-based approach for human body tracking in a diverse set of exercises typically used in musculoskeletal telerehabilitation (and conventional) sessions. Furthermore, this work provides an in-depth description of novel methodologies for coordinate system definition, Range of Motion (ROM) calculation, and data alignment between acquisition systems with different frame rates.

The remainder of the work is structured into the following segments. Section 2 describes the background and main concepts of 2D camera-based systems, drawing conclusions about the most suitable model for the purpose (MediaPipe Pose). Section 3 details the experimental study conducted to evaluate the MediaPipe Pose model for 3D body tracking from monocular video feeds. Section 4 summarizes the main experimental results. Section 5 provides the discussion of the study outcomes. Section 6 draws the main findings and conclusions about this work.

## 2. Background

**Figure 1:**
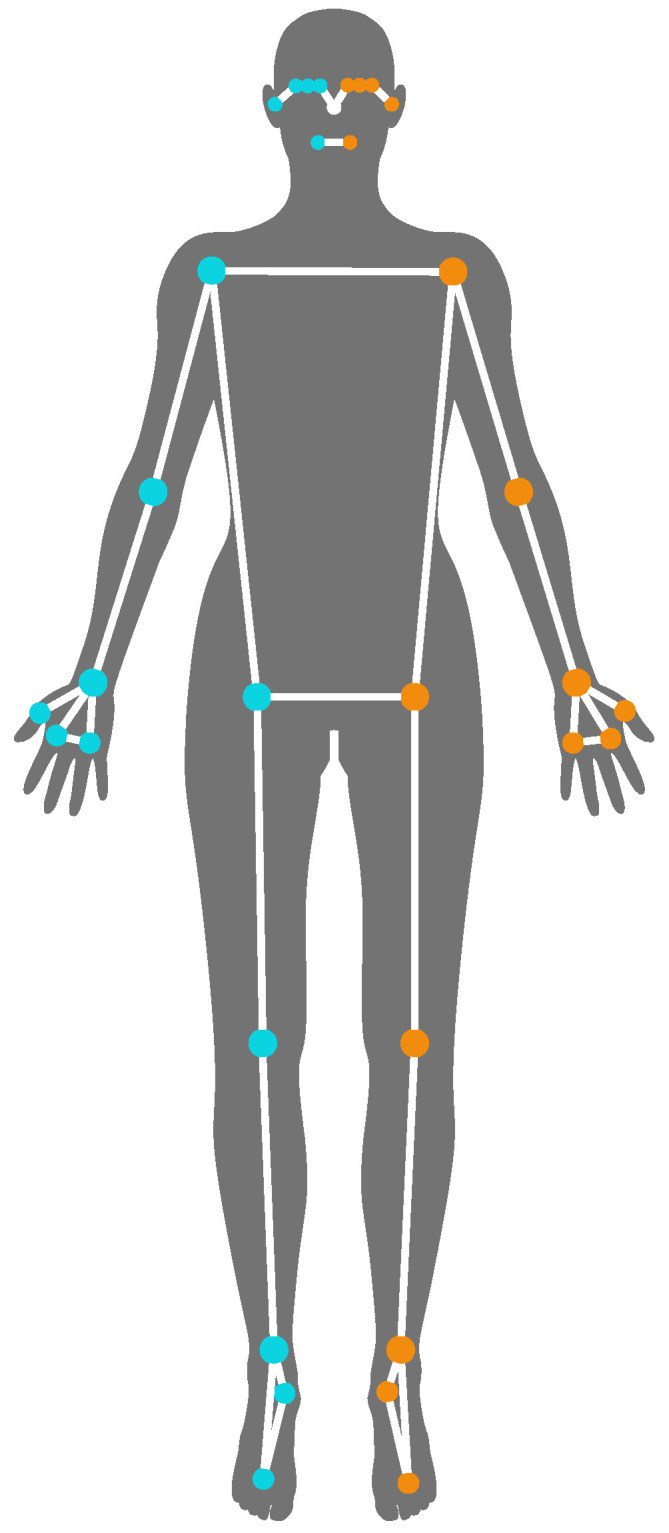
Example of skeletal human body representation: 33 landmarks of MediaPipe Pose, where the right-side landmarks are represented in blue, the left-side landmarks in orange, and the nose landmark in white.

In the context of 2D camera-based systems using deep learning algorithms, human body tracking is commonly referred to as Human Pose Estimation (HPE), and the human body is typically represented by a skeleton model—a tree-like structure composed of landmarks (human joints and other keypoints) connected by edges (body segments) [12], as shown in Figure 1. The joints include shoulders, elbows, wrists, hips, knees, and ankles. Keypoints from the vertebral column, hands, feet, and face may also be integrated into the pose estimation. This body representation uses relatively low-dimensional parameters and is highly valuable for motion capture techniques [12].

**Figure 2:**
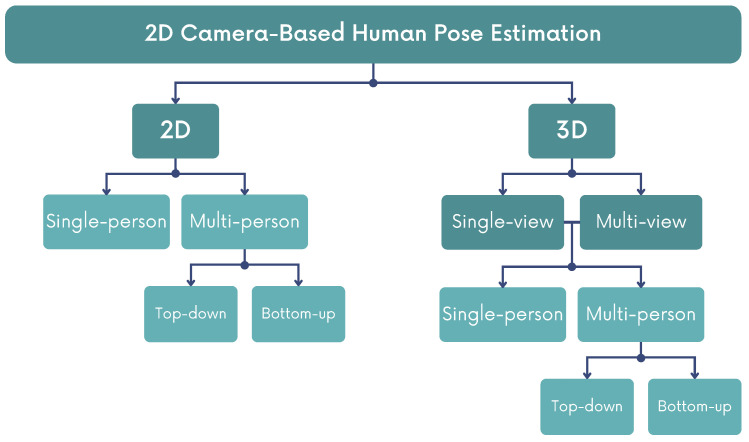
Classification of 2D camera-based models for Human Pose Estimation (HPE).

HPE can be classified based on various criteria (Figure 2), and models can be categorized into 2D HPE or 3D HPE. The former calculates the 2D coordinates (*x*, *y*) of human keypoints, while the latter determines the 3D coordinates (*x*, *y*, *z*) of human joints. The primary difference lies in the depth coordinate (*z*). Additionally, 3D HPE can be classified as single-view (or monocular), when a single 2D camera is used in a fixed position, or as multi-view, when two or more 2D cameras are used to observe the person from different viewpoints [12]. When considering the number of people detected, algorithms can be categorized as single-person or multi-person. Single-person approaches involve identifying the keypoints of a single body in the image or video, while multi-person approaches require the model to identify multiple bodies and their respective landmarks, becoming a challenging task. Multi-person approaches are further divided into top-down and bottom-up methods. Top-down methods first detect individual subjects and then estimate the keypoints (and poses), while bottom-up methods first localize body parts across the image and then associate them to assemble complete human poses. Bottom-up approaches tend to have faster inference, however, top-down methods yield higher accuracy [12].

This work is focused on single-view (or monocular) and single-person approaches. Firstly, monocular approaches require a single camera (fewer resources); furthermore, in telerehabilitation, the treatment sessions are commonly patient-centered, meaning that the camera needs to detect a single subject (single-person approach).

The emergence of deep learning techniques has significantly enhanced the performance of the models for 2D HPE; 3D HPE approaches arise as an extension of 2D HPE [12]. Two of the main challenges of HPE methods are occlusions and rare poses, due to limited training data [12,17]. The sources of occlusions are the person (self-occlusions), other people (in multi-person detections), or external objects [18]. In 2D HPE, numerous approaches have been explored to address occlusions, by adapting the architecture of the deep learning algorithm [12]. In 3D HPE, occlusions can be addressed by collecting information from different viewpoints (a multi-view approach), as an occluded segment in one view may become visible from alternative perspectives [12]. For this work, multi-view approaches are not explored, as the primary goal is to use a single 2D camera for human body tracking.

The performance of 2D HPE has been researched in the literature [12] and various open-source models have been developed, such as PoseNet [19], MoveNet [20], AlphaPose [21], OpenPose [22], and MediaPipe Pose or BlazePose [23]. The evaluation of these models for rehabilitation-related purposes shows encouraging results [24,25,26,27]. Three-dimensional HPE models in the literature include VNect [28], XNect [18], and MediaPipe Pose [23,29]. Although other algorithms have been evaluated in previous research [12], their implementations are typically obfuscated and are rarely evaluated in a physiotherapy context [16].

Considering the aim of this project, MediaPipe Pose was selected; it is a deep learning-based model for 3D HPE from monocular 2D videos with promising performance that combines fast performance, reasonable accuracy, and accessibility [23,29]. MediaPipe Pose predicts the 3D central position of the human joints (Figure 1), in meters, which may be used for several purposes, including calculating the Range of Motion (ROM) of the human joints. This metric is extremely valuable in musculoskeletal physiotherapy since physiotherapists use it to assess the mobility and functioning of joints, and monitor the patient’s evolution [7]. Furthermore, this parameter evaluates pose accuracy independently from scale and body proportions [30].

## 3. Materials and Methods

### 3.1. Experimental Design

**Figure 3:**
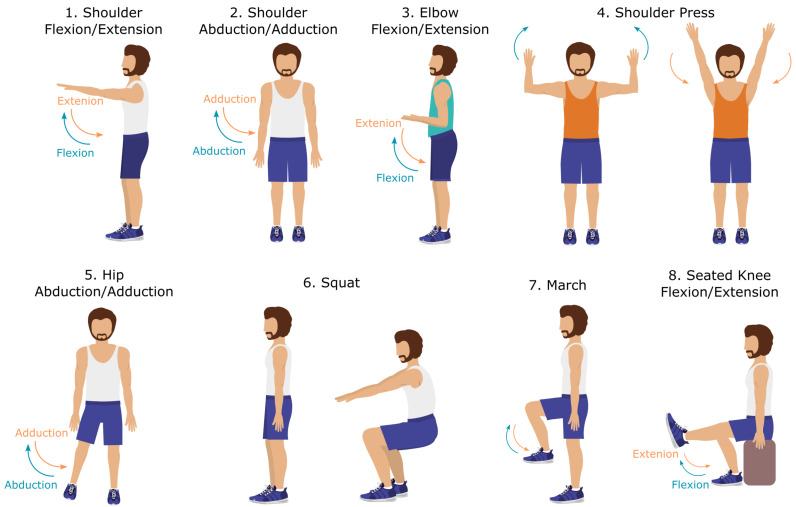
Eight exercises selected for the experimental study: Shoulder Flexion/Extension (SF), Shoulder Abduction/Adduction (SA), Elbow Flexion/Extension (EF), Shoulder Press (SP), Hip Abduction/Adduction (HA), Squat (SQ), March (MCH), and Seated Knee Flexion/Extension (SKF). Shoulder press and squat exercises are illustrated by a sequence of two representative images of the movement.

An experimental study was designed to evaluate the performance of MediaPipe Pose on a wide range of exercises typically performed in musculoskeletal physiotherapy sessions, by comparing it to a gold standard motion tracking system, namely the Qualisys Motion Capture system (Gothenburg, Sweden; https://www.qualisys.com/, accessed on 3 August 2023). Eight exercises were selected: Shoulder Flexion/Extension (SF), Shoulder Abduction/Adduction (SA), Elbow Flexion/Extension (EF), Shoulder Press (SP), Hip Abduction/Adduction (HA), Squat (SQ), March (MCH), and Seated Knee Flexion/Extension (SKF). The exercises are described in Table 1 and shown in Figure 3. The exercise selection was based on their engagement of diverse joints (shoulder, elbow, hip, and knee), body poses, moving limbs (upper and lower limbs), and planes of movement (frontal and sagittal). A broad range of movements allows the evaluation of the model given different conditions, each presenting various challenges in diversity, complexity, and occlusions.

Our study involved eight healthy participants (seven females and one male) aged between 19 and 21 years old. In order to assess the physical condition and musculoskeletal health of the participants, a questionnaire was designed to gather relevant information, including neuro-musculoskeletal injuries clinically diagnosed in the last three months, movements that elicited pain, and any previous musculoskeletal surgeries. All subjects were deemed eligible and were enrolled in the study. The experimental protocol was approved by the Ethics Committee of the Escola Superior de Saúde de Santa Maria (Reference: CE2022/09). A written informed consent was obtained from all participants.

### 3.2. Experimental Data Acquisition

**Figure 4:**
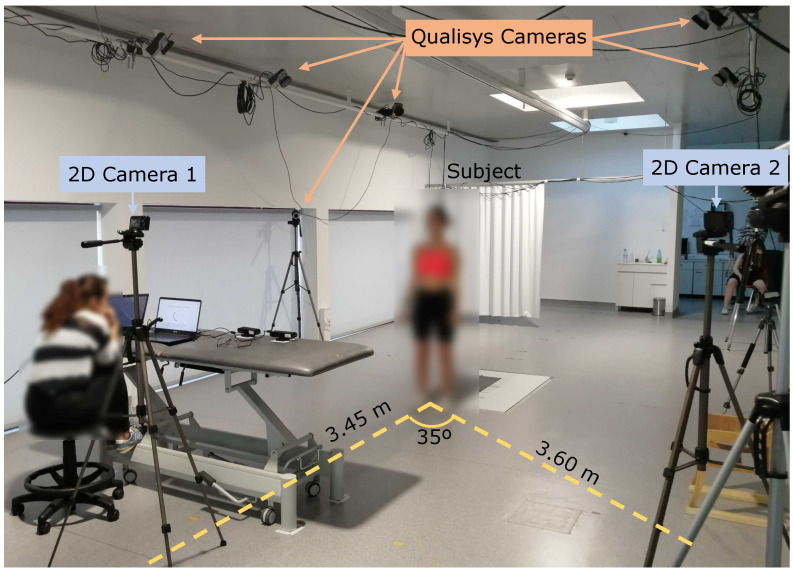
Experimental setup for the data acquisition, showing some of the Qualisys cameras, two 2D cameras, and the relative position between the subject and the two 2D cameras.

Experimental data acquisition was conducted in the biomechanics laboratory of the Centro de Investigação em Reabilitação (CIR) at the Escola Superior de Saúde do Instituto Politécnico do Porto. Healthy participants performed eight exercises displayed on a screen to guide them through the execution of the movements. Before initiating each exercise, a preview of the movement was shown to illustrate the exercise that the participants were expected to perform. Then, the acquisition consisted of each participant performing two sets of seven exercise repetitions, with a 10-second resting period between sets. Between acquisitions, participants were advised to rest by sitting on a chair before starting the following exercise. A total of 64 acquisitions (eight participants × eight exercises) were collected, of which only 63 were studied due to a technical issue during the shoulder flexion exercise performed by Subject 5. For each acquisition, the following data were collected simultaneously: (1) ground truth data, i.e., 3D coordinates (*x*, *y*, *z*) of various anatomical landmarks, using the Qualisys Motion Capture system (recording data at 100 frames per second, FPS) from the laboratory; and (2) monocular 2D video recordings, using a Nikon Coolpix A10 camera (operating with 1280 × 720 resolution at 30 FPS). Figure 4 depicts the experimental setup.

**Figure 5:**
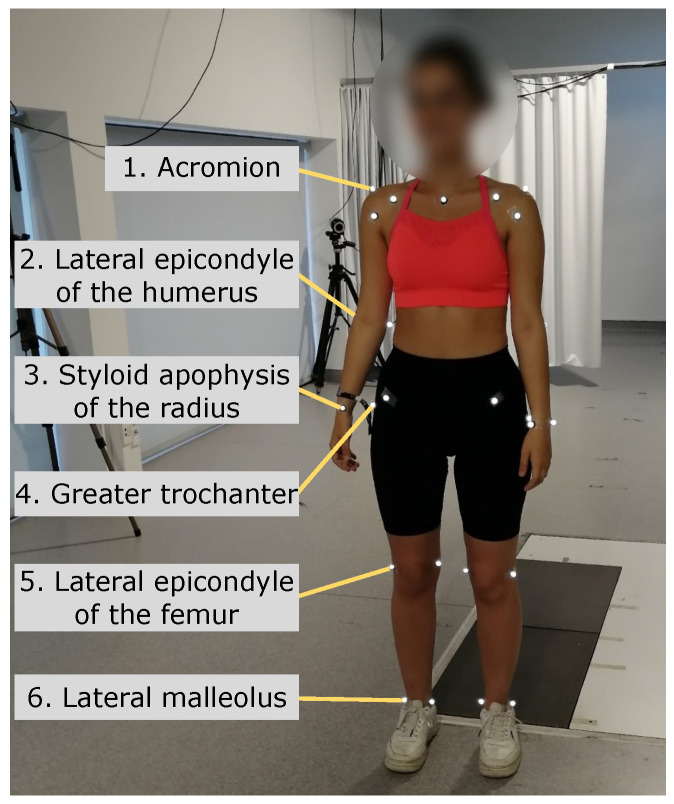
Anatomical location of the six Qualisys MoCap markers.

Before starting the data acquisition, the Qualisys configuration involved the calibration of the 12 infrared cameras, followed by the placement of motion capture (MoCap) markers on specific anatomical regions of the participants. For this study, data from only six MoCap markers placed on the right side of the body were required. It was essential to determine and establish the correct position of the anthropometric points, as these were used as ground truth measurements; therefore, the anatomical points of the MoCap markers were defined and confirmed by a physiotherapist researcher experienced in palpatory anatomy. Figure 5 shows the anatomical location of the markers, and Table 2 describes the association between the anatomical location of the six Qualisys MoCap markers and the human joints. For the 2D video recording, two identical 2D cameras were used (only one was operating at any one time); 2D camera 1 recorded frontal plane exercises and was parallel to the participant’s frontal plane, and 2D camera 2 recorded sagittal plane exercises and was positioned at an angle of 35° to the participant’s frontal plane (Figure 4). The previous information describes the camera position that minimizes the number of occlusions during the exercise execution.

### 3.3. Data Preprocessing

The 2D videos from the experimental acquisition were given as input for the MediaPipe Pose model to estimate the 3D coordinates of the human joints. The raw data from the Qualisys system and the MediaPipe Pose model consisted of the 3D positions of the MoCap markers and the 3D central positions of the joints, respectively. For the ROM evaluation, both data sources provide approximately equivalent information after converting the 3D positions into amplitude values. Additionally, proper alignment was necessary for comparing ground truth and predicted values from the same time point. The procedures are described next.

#### 3.3.1. 3D Cartesian Coordinate System

For physiotherapy purposes, defining a 3D coordinate system coincident with the normal vectors of the anatomical planes is valuable information, particularly when determining the joint amplitude or Range of Motion (ROM).

**Figure 6:**
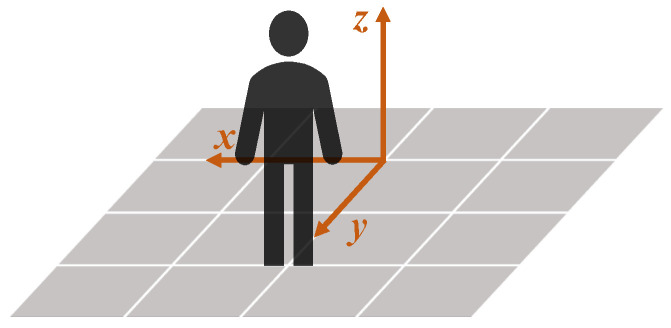
The 3D Cartesian coordinate system of Qualisys (in orange) and its spatial relation with respect to the participant position during data acquisition.

**Figure 7:**
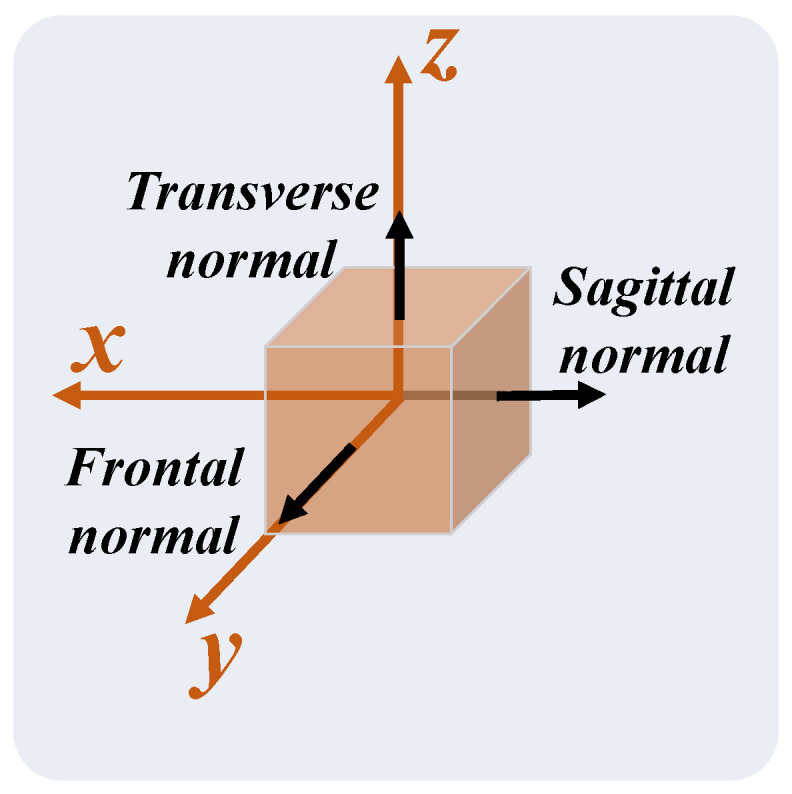
Comparison of the normal vectors of the anatomical planes (in black) with the Qualisys coordinate system (in orange).

The Qualisys coordinate system is shown in Figure 6. The direction of the axes of the Qualisys coordinate system was assumed to be parallel to the normal vectors of the anatomical planes of the participant (Figure 7).

**Figure 8:**
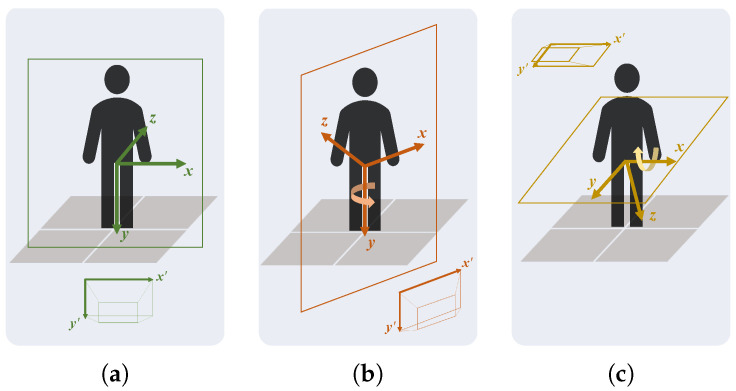
Relation between the participant position and the Cartesian coordinate system of the MediaPipe Pose model for three camera orientations: (**a**) camera plane parallel to participant frontal plane; (**b**) camera plane rotated around the Y-axis relative to participant frontal plane; and (**c**) camera plane rotated around the X-axis relative to participant frontal plane. The camera 2D coordinate system is represented by the X’-axis and Y’-axis, which are parallel to the X-axis and Y-axis of the algorithm coordinate system, respectively.

**Figure 9:**
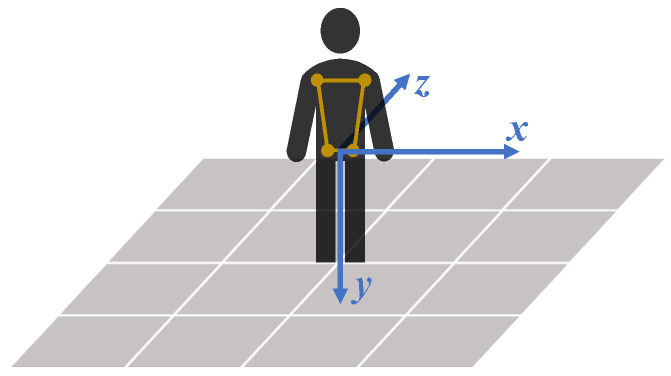
The virtual 3D coordinate system of MediaPipe Pose coincident with the normal vectors of the anatomical planes. The origin is the midpoint between the hips. The X-axis is the sagittal plane normal, the Y-axis is the transverse plane normal, and the Z-axis is the frontal plane normal. The four points (representing the shoulders and hips) are used to define the virtual 3D coordinate system.

The MediaPipe Pose coordinate system depends on the relative position between the 2D camera and the participant, as illustrated in Figure 8. The origin is the midpoint between the hips; the XY plane of the MediaPipe Pose coordinate system is parallel to the X’Y’ plane of the camera plane. The Z-axis is the third direction according to the right-hand rule. Due to the camera position dependency, no direct relationship between the MediaPipe Pose and anatomical coordinate systems can be assumed. Therefore, a virtual coordinate system was defined coincident with the anatomical planes, such that the Z-axis is the normal vector to the frontal plane, the X-axis the normal vector to the sagittal plane, and the Y-axis the normal vector to the transverse plane (Figure 9).

**Figure 10:**
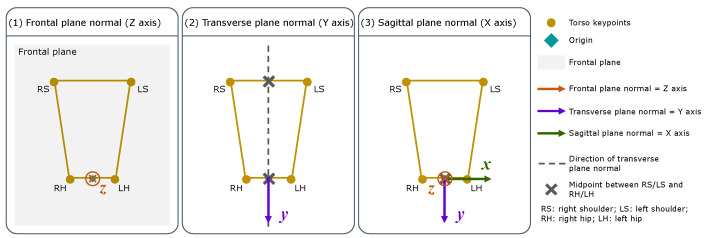
Representation of virtual 3D coordinate system definition: (1) Z-axis or frontal plane normal; (2) Y-axis or transverse plane normal; and (3) X-axis or sagittal plane normal.

**Figure 11:**
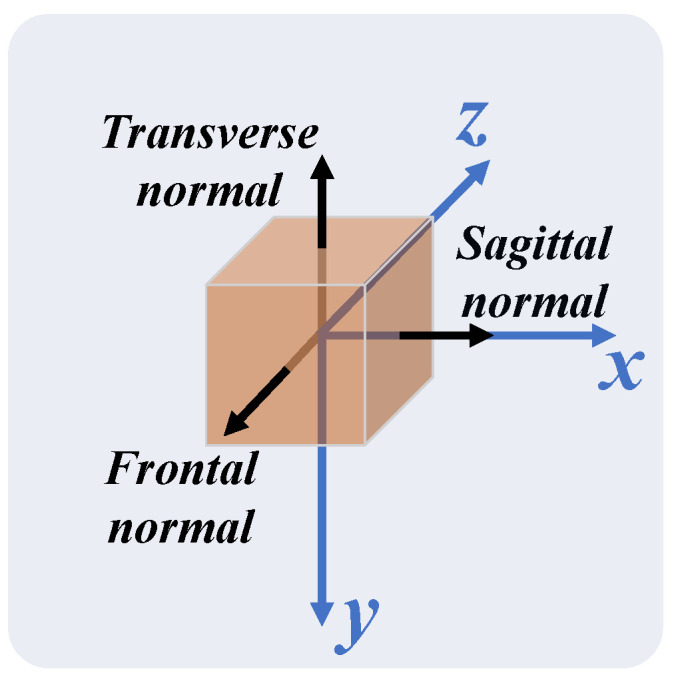
Comparison of the normal vectors of the anatomical planes (in black) with the MediaPipe Pose virtual coordinate system (in blue).

Figure 10 shows the three main steps to define the virtual coordinate system. The origin is the midpoint between the hips, and the direction of the axes was defined using the torso joint positions (shoulders and hips) estimated by MediaPipe Pose in the first frame. Firstly, the frontal plane normal (Z-axis) was defined as the normal vector to the best plane containing the four torso keypoints, using the RANSAC regressor [31]. Secondly, the transverse plane normal (Y-axis) was defined as the vector from the shoulders’ midpoint to the hips’ midpoint. Lastly, the sagittal plane normal (X-axis) was calculated using the right-hand rule. The direction of the axes of the MediaPipe Pose virtual coordinate system was assumed to be parallel to the normal vectors of the anatomical planes of the participant (Figure 11).

#### 3.3.2. Amplitude Calculation

**Figure 12:**
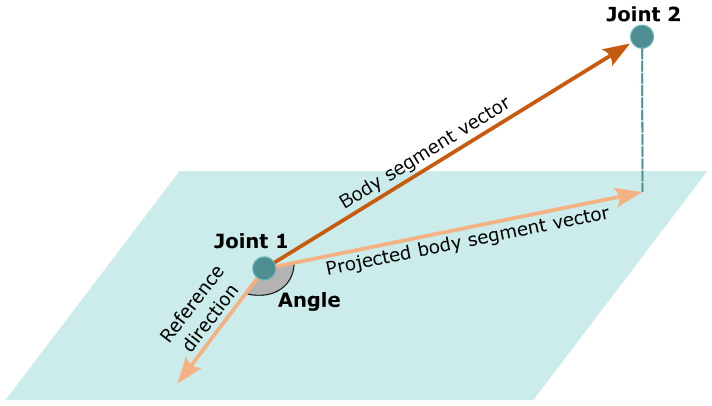
Amplitude calculation between the projected body segment vector and a reference direction.

In musculoskeletal physiotherapy, the Range of Motion (ROM) is defined according to the neutral-zero method; the ROM is determined by moving the distal segment of a joint from a neutral starting position (zero position) to the end position around a rotation axis [7]. Therefore, the joint amplitude is the angle between the body segment (projected in the plane of movement) and a reference direction (zero position), as shown in Figure 12. The body segment is the vector between two joints: the joint where the ROM is being evaluated and the closest joint to the evaluated joint in the moving limb. The projection of the body segment of the plane of movement ensures that only the angle component of that plane is being investigated. For instance, for the shoulder abduction/adduction exercise, only the frontal plane component assesses the shoulder joint on abduction/adduction motions; the sagittal plane component evaluates it on flexion/extension motions. The normal to the plane of movement is the only information necessary for the projection of the body segment on that plane. The reference direction is the vector with respect to which the amplitude is defined, meaning that it corresponds to the 0° amplitude (zero position). The information for the amplitude calculation for each exercise is shown in Table 3.

#### 3.3.3. Data Alignment

**Figure 13:**
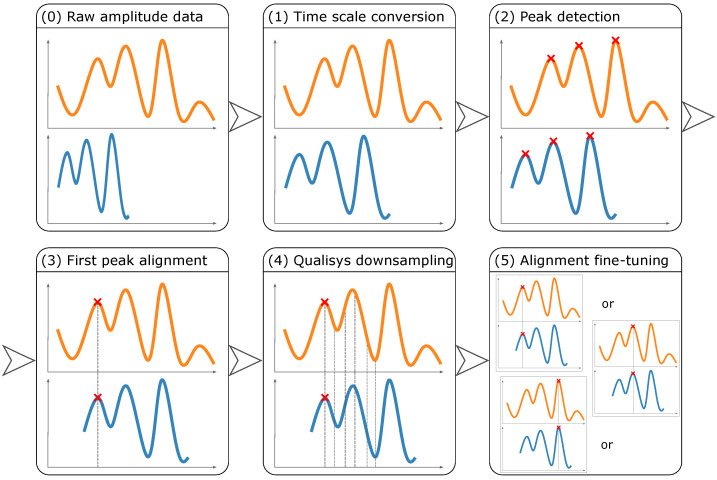
Data alignment between the Qualisys ground truth amplitudes (in orange) and MediaPipe Pose predicted amplitudes (in blue).

After determining the raw amplitude data, finding matching time points between Qualisys and MediaPipe Pose amplitudes was required. This was achieved by implementing five steps (Figure 13): (1) converting frames into a time scale, in seconds, knowing the frame rate of Qualisys (100 FPS) and 2D videos (30 FPS); (2) finding maximum (peak) amplitudes, and the respective time points; (3) aligning amplitude acquisitions by the first peak time point; (4) downsampling Qualisys time points by selecting the ones that matched MediaPipe Pose time points; and (5) fine-tuning the alignment by selecting the pair of peak amplitudes (one from Qualisys and the other from MediaPipe Pose) that yielded the highest Pearson correlation coefficient between them.

Since each exercise was repeated multiple times during a single acquisition, segmentation was performed to extract the exercise repetitions from the recordings. Data segments corresponding to resting periods, exercise familiarization, or uncompleted trials were not considered repetitions.

### 3.4. Evaluation Metrics

A comprehensive analysis of the performance of the MediaPipe Pose model in amplitude prediction was conducted by comparing the model amplitudes with the ground truth amplitudes for each exercise across all participants. Firstly, two error metrics were selected to assess the model accuracy: Mean Absolute Error (MAE) and Mean Absolute Percentage Error (MAPE), defined by Equations (1) and (2), respectively:

(1) $$\mathrm{MAE}=\frac{1}{n}\sum_{i=1}^{n}\left|y_{i}-{\hat{y}}_{i}\right|$$

(2) $$\mathrm{MAPE}=\frac{1}{n}\sum_{i=1}^{n}\left|\frac{y_{i}-{\hat{y}}_{i}}{y_{i}}\right|\times 100$$

where *n* is the number of observations (i.e., the number of collected frames for each exercise), $y_{i}$ is the ground truth value for observation *i*, and ${\hat{y}}_{i}$ is the predicted value for observation *i*. Then, to quantify the correlation between two variables (Qualisys ground truth amplitudes and MediaPipe Pose predicted amplitudes), two metrics were evaluated: Pearson correlation coefficient and cosine similarity. Pearson correlation coefficient measures the linear relationship between two sets of data points and takes into account both their magnitude and direction, meaning that it is sensitive to the scale of the data [32]. Additionally, cosine similarity measures similarity as the cosine of the angle between two vectors [33]. Unlike Pearson correlation, this metric is not sensitive to the magnitude of data, only to their direction, meaning that it assesses the morphology of the acquisition without considering its scale. Finally, the properties of a linear regression between the Qualisys and MediaPipe Pose data were investigated.

## 4. Results

**Figure 14:**
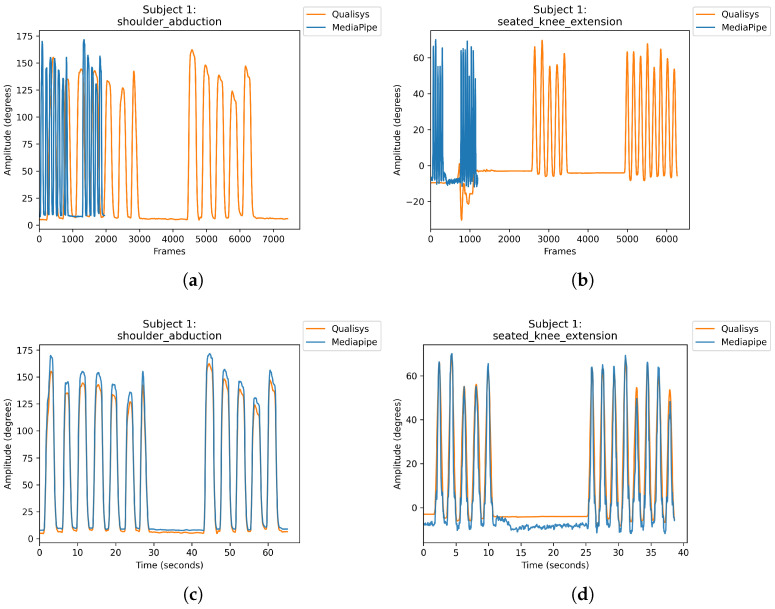
Example of Qualisys ground truth (in orange) and MediaPipe Pose predicted (in blue) amplitudes for Subject 1 performing SA exercise and SKF exercise. (**a**,**b**) show the raw amplitude before the alignment procedure, and (**c**,**d**) the aligned amplitude data, before segmenting the sample to extract the exercise repetitions.

For the ROM study, results consisted of pairs of ground truth amplitudes measured by Qualisys and amplitudes predicted by MediaPipe Pose collected from eight participants performing eight exercises frequently performed in physiotherapy sessions. Figure 14 shows representative traces of Qualisys ground truth (in orange) and MediaPipe Pose predicted (in blue) amplitudes over time during the SA and SKF exercises performed by Subject 1, highlighting the raw amplitude data before the alignment procedure (Figure 14a,b) and the aligned amplitude data (Figure 14c,d).

The evaluation of the MediaPipe Pose model for ROM estimation was conducted from two perspectives: peak amplitudes and motion amplitudes. Peak amplitudes represent the maximum articular angle measured in each exercise repetition. It is an important parameter for physiotherapists to assess their patients’ progress during treatment [7]. Moreover, investigating all amplitude predictions during exercise execution (motion amplitudes) is also important, as it can provide a comprehensive evaluation of the algorithm’s performance for a wider range of angles.

MAE and MAPE (Equations (1) and (2)) for both peak and motion amplitudes are shown in Table 4. MAPE evaluation was based on previously used criteria [34], and different colors were used to highlight various performances (Table 4). For the peak amplitude analysis, MAE varied from 3.7° (HA) to 28.8° (SF), and MAPE varied from 6.6% (SKF) to 28.7% (SF). Both MAE and MAPE were higher for upper-body exercises than for lower-body exercises. For most exercises, the model had a MAPE below or close to 10% (highly accurate forecasts), suggesting promising results. For motion amplitudes, absolute angles between 0° and 1° were eliminated (threshold = 1°) to prevent infinite errors. MAE varied from 3.2° (HA) to 18.7° (SP), and MAPE between 14.9% (SA) and 107.4% (MCH). SA, SP, EF, and SQ exercises showed the lowest MAPE, while MCH, SKF, SF, and HA exercises showed the highest MAPE.

The results to quantify the correlation between two variables (Qualisys ground truth amplitudes and MediaPipe Pose predicted amplitudes) are shown in Table 5. For peak amplitudes, the Pearson coefficient varied between 0.744 (SP) and 0.961 (MCH). When Pearson coefficients are above 0.9 (as shown in SA, EF, HA, and MCH), the association between ground truth and predicted data is considered very strong [35]. The *p*-value was <0.001, indicating a statistically significant Pearson coefficient [32]. Cosine similarity was equal to or greater than 0.992 for all exercises, suggesting a strong relationship between data morphology from Qualisys and MediaPipe Pose, regardless of their magnitudes. For motion amplitudes, the Pearson coefficient was equal to or greater than 0.904 (very strong correlation [35]) for all exercises; cosine similarity values were equal to or greater than 0.940. Similarly to the MAPE study of motion amplitudes, frontal plane upper-body exercises (SA and SP), EF, and SQ exercises were the ones with the highest cosine similarity value (above 0.990).

**Figure 15:**
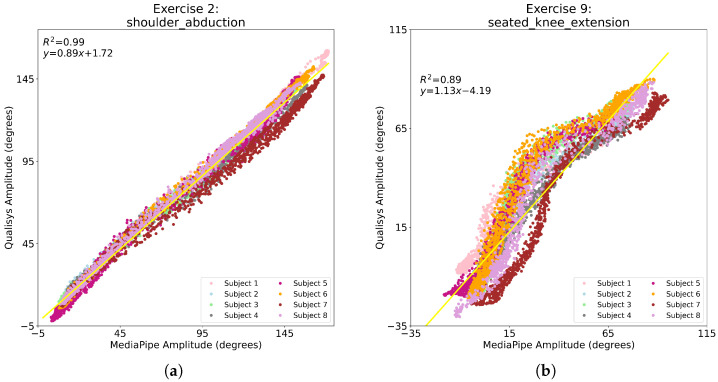
Relation between Qualisys and MediaPipe Pose motion amplitudes for (**a**) SA exercise and (**b**) SKF exercise. Each color represents a different subject, and the yellow line is the linear regression that best fits the amplitude data for the exercise; the coefficient of determination ($R^{2}$) and the linear regression equation (slope and intercept) are also shown, where *y* and *x* are the Qualisys and MediaPipe Pose amplitudes, respectively.

Amplitudes measured by Qualisys and predicted by MediaPipe Pose were displayed on plots to visualize the relationship between ground truth and predictions, demonstrated by the high Pearson coefficient and cosine similarity seen in Table 5. Representative plots of the relationship between Qualisys and MediaPipe Pose motion amplitudes for SA and SKF exercises are shown in Figure 15. Results for all exercises are in Table 6. The coefficient of determination ($R^{2}$) assesses how well the linear regression fits the data. $R^{2}$ values ranged between 0.82 (SF) and 0.99 (SA). $R^{2}$ values were the highest for the frontal plane (SA, SP, HA) and SQ exercises. MCH and EF also showed high $R^{2}$, while SKF and SF were the exercises with the lowest $R^{2}$ results. Frontal plane exercises, SQ, and MCH plots showed an approximately linear relationship between data (Figure 15a), and SF, SKF, and EF showed an approximately cubic polynomial relationship between data (Figure 15b).

## 5. Discussion

Error and correlation analyses provided valuable insights about the MediaPipe Pose performance. Firstly, the model predictions for peak amplitudes showed low MAE and MAPE, indicating a promising performance of MediaPipe Pose, in particular for lower-body exercises, where the ROM is commonly lower than in upper-body exercises. Secondly, the model predictions for motion amplitudes showed higher MAE and MAPE than for peak amplitudes, suggesting that the algorithm performed better in static poses (peak amplitudes) than in dynamic movements (motion amplitudes) [35]; furthermore, MediaPipe Pose showed lower MAPE for frontal plane upper-body (SA and SP), EF, and SQ exercises than for HA, SF, MCH, and SKF exercises.

SA is a frontal plane, upper-body, and unilateral exercise. The model’s performance for frontal plane exercises was expected to be better than for sagittal plane exercises since, for the frontal configuration, the movement occurs in a plane approximately parallel to the camera plane; thus, all joints are approximately at the same angle to the camera (same depth). Depth ambiguities, mainly associated with side-views where different depths need to be determined for each joint, are a primary challenge in monocular 3D HPE [36], which are avoided in frontal plane exercises. Besides depth ambiguities, other factors influence the 3D estimations, justifying why other frontal plane exercises (SP and HA) did not show performance as good as the SA exercise. For instance, training data with a lack of representative examples for movements, such as SP and HA, may also make the prediction by the algorithm more challenging [37].

For motion amplitudes, EF is a sagittal plane, upper-body, and unilateral exercise with one of the lowest MAPE. In this exercise, only the right forearm (i.e., right wrist joint) was moving, and during the exercise execution, this body segment was not occluded. The low MAPE values for motion amplitudes suggested that MediaPipe Pose can correctly predict the right elbow and wrist (only joints considered for this exercise). On the other hand, SKF was the exercise with one of the highest MAPE. This exercise involves unusual poses and self-occlusions that may be challenging for MediaPipe Pose. This is likely due to the lack of training data representing those poses and self-occlusions [17,37].

SQ and MCH exercises are more prone to self-occlusions and unusual poses since their execution involves moving several joints simultaneously. Interestingly, SQ was one of the exercises with the lowest amplitude error (MAPE), probably because the right knee, hip, and ankle are the only joints evaluated in the ROM analysis and these are visible (not occluded) during the exercise execution. The MCH exercise is a bilateral movement, where each leg moves one at a time. Although only the right leg (further from the camera) motion was evaluated, the motion of the left leg (closer to the camera) in front of the right leg (self-occlusion) may contribute to a high amplitude error (MAPE) [17].

In summary, MediaPipe Pose errors reported previously may be due to depth ambiguities [38], self-occlusions [17], or challenging poses [39]. Furthermore, erroneous 2D estimations may also affect the 3D HPE [40]; MediaPipe Pose estimates the 3D positions from the predicted 2D positions (*2D to 3D lifting* technique). 3D HPE models may incorporate *2D to 3D lifting* techniques, where an intermediate 2D pose is first estimated, and then lifted to 3D, i.e., estimate the 3D position of the joints from their 2D positions, meaning that higher 2D errors may contribute to higher 3D errors. The amount of annotated data can also influence deep learning algorithms; wider variability of scenarios (unusual poses and occlusions) present in the training dataset has been found to contribute to a better performance of these models [41]. The MediaPipe Pose model was trained on a customized dataset [23,29], capturing a wide range of fitness poses. Nevertheless, some poses from the selected exercises may not be widely represented in its dataset, making the model prediction harder. Additionally, the experimental data acquisition by the Qualisys system [42,43], as well as data preprocessing oversimplification when converting 3D joint positions into amplitude values, may introduce errors in the ground truth data.

For motion amplitudes, despite the high MAPE results seen in some exercises (as high as 107.4%), a strong correlation between Qualisys ground truth and MediaPipe Pose predicted amplitudes was observed (Pearson coefficient and cosine similarity above 0.9) [35]. Taken together, MAPE and correlation results seemed to indicate that MediaPipe Pose predictions replicated the shape of the curve of the Qualisys data (high correlation), but shifted or scaled (high MAPE). Therefore, for the frontal plane, SQ and MCH exercises, the linear function shown in Table 6 could be used to map MediaPipe Pose predictions into Qualisys ground truth amplitudes, in order to decrease the error between them. Similarly, a fine-tuned cubic polynomial function could be applied to SF, EF, and SKF exercises [44].

Overall, the exercise with the best results was SA, a frontal plane, upper-body, and unilateral exercise, while the exercises with the lowest performance were the MCH and SKF, where complex poses and numerous occlusions can be found, and SF, where the depth may be harder to estimate.

## 6. Conclusions

This work explored the potential of using approaches for 3D HPE from monocular 2D video feeds in musculoskeletal telerehabilitation. Specifically, the performance of MediaPipe Pose was evaluated on a wide range of exercises commonly performed in physiotherapy sessions, covering different body poses. The investigation of MediaPipe Pose for 3D HPE was focused on joint ROM, the metric used by physiotherapists in both telerehabilitation and conventional sessions to follow patients. As a valuable addition to existing research, this study also provided an in-depth description of (1) a novel approach for defining a 3D Cartesian coordinate system, invariant to camera orientation, suitable for both 2D and 3D camera acquisitions; (2) the calculation of the Range of Motion (ROM), considering the physiotherapy definition; and (3) the description of the data alignment procedure for acquisition systems with different frame rates.

The MediaPipe Pose model yielded promising results, with the SA, SP, EF, and SQ exercises generally showing better performance than the remaining exercises. Overall, the exercise with the best results was SA, a frontal plane, upper-body, and unilateral exercise, while the exercises with the lowest performance were the MCH, SKF, and SF.

In conclusion, this study supports the potential of using MediaPipe Pose for 3D body tracking from monocular 2D videos in musculoskeletal telerehabilitation applications, to eliminate the need for complex specialized hardware, such as 3D depth cameras or wearables. Although the results varied under different conditions, the MediaPipe Pose model showed encouraging performance. Future work should include testing other state-of-the-art algorithms, increasing the sample size of participants in the study, extending the dataset to subjects with musculoskeletal diseases, investigating post-preprocessing techniques to enhance the results, and gathering additional training data focused on physiotherapy-specific motions (such as the Fit3D dataset [45]) and poses to handle challenges, such as occlusions and depth ambiguities. With further refinements to the models, 3D body tracking from monocular 2D video feeds appears to be a viable, affordable, and accessible approach for musculoskeletal telerehabilitation solutions. In the future, this can help the development of better physiotherapy options for patients with musculoskeletal disorders.

## Figures and Tables

**Table 1:** Exercises commonly performed in musculoskeletal physiotherapy sessions, and description of the limb in motion, the plane of movement, and the evaluated joint.

Exercises	Limb in Motion	Plane of Movement	Evaluated Joint
1. Shoulder Flexion/Extension (SF)	Right arm	Sagittal	Right shoulder
2. Shoulder Abduction/Adduction (SA)	Right arm	Frontal	Right shoulder
3. Elbow Flexion/Extension (EF)	Arms (bilateral)	Sagittal	Right elbow
4. Shoulder Press (SP)	Arms (bilateral)	Frontal	Right shoulder
5. Hip Abduction/Adduction (HA)	Right leg	Frontal	Right hip
6. Squat (SQ)	Legs (bilateral)	Sagittal	Right knee
7. March (MCH)	Legs (bilateral)	Sagittal	Right hip
8. Seated Knee Flexion/Extension (SKF)	Right leg	Sagittal	Right knee

**Table 2:** Relation between the anatomical location of the six Qualisys MoCap markers and the human joints.

MoCap Anatomical Location	Joint
1. Acromion	Shoulder
2. Lateral epicondyle of humerus	Elbow
3. Styloid apophysis of radius	Wrist
4. Greater trochanter	Hip
5. Lateral epicondyle of the femur	Knee
6. Lateral malleolus of the ankle	Ankle

**Table 3:** Information for the ROM evaluation: plane of movement in which the exercise occurs, the body segment, and the reference direction. ↓ represents vertically downward direction.

Exercises	Plane of Movement	Body Segment (Joint 1–Joint 2)	Reference Direction
1. SF	Sagittal	Shoulder–elbow	↓
2. SA	Frontal	Shoulder–elbow	↓
3. EF	Sagittal	Elbow–wrist	↓
4. SP	Frontal	Shoulder–elbow	↓
5. HA	Frontal	Hip–knee	↓
6. SQ	Sagittal	Knee–hip	Foot-knee
7. MCH	Sagittal	Hip–knee	↓
8. SKF	Sagittal	Knee–foot	↓

**Table 4:** MAE, in degrees, and MAPE, in percentage, between Qualisys and MediaPipe Pose amplitudes (peak and motion) for each exercise. MAPE color code [34]: <10% (highly accurate forecast) in green; 10–20% (good forecast) in yellow; 20–50% (reasonable forecast) in light orange; >50% (inaccurate forecast) in dark orange. (s) and (f) indicate sagittal and frontal plane exercises, respectively.

Exercise	Peak Amplitudes		Motion Amplitudes (Threshold = 1°)	
	**MAE (°)**	**MAPE (%)**	**MAE (°)**	**MAPE (%)**
1. SF (s)	28.8	28.7	15.6	66.60
2. SA (f)	13.0	10.2	7.7	14.90
3. EF (s)	11.7	9.6	10.6	24.2
4. SP (f)	13.8	9.5	18.7	23.0
5. HA (f)	3.7	9.0	3.2	62.9
6. SQ (s)	7.6	7.9	8.3	25.0
7. MCH (s)	6.3	7.7	6.3	107.4
8. SKF (s)	4.9	6.6	9.9	78.10

**Table 5:** Correlation analysis between the Qualysis and MediaPipe Pose amplitudes (peak and motion) for each exercise: Pearson correlation coefficient (*r*) and cosine similarity coefficient (cos_sim). The *p*-value was <0.001, indicating a statistically significant Pearson coefficient. Color code: >0.9 in green; 0.8–0.9 in yellow; 0.7–0.8 in light orange. (s) and (f) indicate sagittal and frontal plane exercises, respectively.

Exercise	Peak Amplitudes		Motion Amplitudes	
	r	**cos_sim**	r	**cos_sim**
1. SF (s)	0.894	0.992	0.904	0.949
2. SA (f)	0.939	0.999	0.996	0.999
3. EF (s)	0.903	0.997	0.963	0.990
4. SP (f)	0.744	0.999	0.985	0.997
5. HA (f)	0.915	0.995	0.985	0.987
6. SQ (s)	0.833	0.998	0.981	0.993
7. MCH (s)	0.961	0.996	0.964	0.979
8. SKF (s)	0.765	0.997	0.942	0.961

**Table 6:** Linear regression between motion amplitudes of Qualisys and MediaPipe Pose for each exercise: slope and intercept values for the equation that better fits the transformation of predictions (MediaPipe Pose points) into expected data (Qualisys points), coefficient of determination ($R^{2}$), and curve shape. (s) and (f) indicate sagittal and frontal plane exercises, respectively.

Exercise	Motion Amplitudes			
	**Slope**	**Intercept**	$R^{2}$	**Curve Shape**
1. SF (s)	0.75	11.3	0.82	Not linear
2. SA (f)	0.89	1.72	0.99	Linear
3. EF (s)	1.23	−11.86	0.93	Not linear
4. SP (f)	0.96	−14.2	0.97	Linear
5. HA (f)	0.92	3.39	0.97	Linear
6. SQ (s)	1.05	5.12	0.96	Linear
7. MCH (s)	1.03	−0.82	0.93	Linear
8. SKF (s)	1.13	−4.19	0.89	Not linear

## Data Availability

The data and source code underlying this research are openly available. The dataset of the 3D positions measured by the Qualysis Motion Capture system and the dataset of the 3D positions predicted by the MediaPipe Pose model are available at the following Zenodo repository: https://zenodo.org/records/10408307. The source code is available at the following GitHub repository: https://github.com/carolinaclemente00/3D-HPE-MediaPipe-Pose/.

## Abbreviations

The following abbreviations are used in this manuscript:

HPE	Human Pose Estimation
MoCap	Motion Capture
ROM	Range of Motion
FPS	Frames per Second
SF	Shoulder Flexion/Extension
SA	Shoulder Abduction/Adduction
EF	Elbow Flexion/Extension
SP	Shoulder Press
HA	Hip Abduction/Adduction
SQ	Squat
MCH	March
SKF	Seated Knee Flexion/Extension
MAE	Mean Absolute Error
MAPE	Mean Absolute Percentage Error
